# Emotional maltreatment and problematic short-video use among adolescents: an indirect association through narcissism and moderation of the direct association by physical activity

**DOI:** 10.3389/fpsyt.2026.1958164

**Published:** 2026-09-25

**Authors:** Yuhan Chen, Xiaodi Ma, Ning Wang, Geng Li, Dan Liang, Anfa Yuan, Lei Xiang, Shiqi Long, Jianzhang Luo, Yang Liu

**Affiliations:** 1 School of Sports Science, Jishou University, Jishou, China; 2 Heilongjiang University of Chinese Medicine, Harbin, China; 3 School of Physical Education, Chengdu Vocational University of the Arts, Chengdu, China; 4 School of Psychology, Research Center for Exercise and Brain Science, Shanghai University of Sport, Shanghai, China; 5 Yulin Normal University, Yulin, China; 6 School of Physical Education, Xichang University, Xichang, China; 7 Hunan Medical College, Huaihua, China

**Keywords:** adolescents, emotional maltreatment, narcissism, physical activity, problematic short-video use

## Abstract

**Introduction:**

Emotional maltreatment has been associated with problematic short-video use during adolescence, but the nature of this association remains unclear. This cross-sectional study examined the association between emotional maltreatment and problematic short-video use, estimated the indirect statistical association through narcissism, and tested whether physical activity moderated the direct association between emotional maltreatment and problematic short-video use.

**Methods:**

Data were obtained in November 2025 from six schools across three Chinese provinces. The analytic sample included 1,622 adolescents, comprising 772 males and 850 females. Participants completed measures of emotional maltreatment, narcissism, problematic short-video use, and physical activity.

**Results:**

Emotional maltreatment was positively associated with problematic short-video use, and an indirect statistical association was observed through narcissism. Physical activity moderated the direct association between emotional maltreatment and problematic short-video use, which was weaker at higher levels of physical activity.

**Discussion:**

Considering a personality characteristic and a health-related behavior within the same model provides a broader understanding of the circumstances under which emotional maltreatment is associated with problematic short-video use. These findings may inform family- and school-based support concerning adolescents’ emotional needs, healthy short-video use, and regular physical activity.

## 1 Introduction

Problematic short-video use (PSVU) refers to persistent and difficult-to-control engagement with short-form video content and may be accompanied by disruptions in daily functioning (1–3). With the rapid expansion of short-video platforms, PSVU has received increasing attention in adolescent health research. Adolescence is a critical developmental period during which self-regulatory capacities remain relatively immature. Against the background of frequent exposure to short-video platforms, this developmental characteristic may be associated with greater susceptibility to problematic patterns of use (4). Such developmental vulnerability warrants particular attention given the widespread use of short-video platforms among younger populations. A review of 26 studies involving 11,462 participants reported an overall TikTok use rate of 80.19%, with particularly high use among younger individuals (5). China has 1.053 billion short-form video users. Meanwhile, the number of adolescent internet users is approaching 200 million, and the reported short-video usage rate among adolescents is 65.3% (6). Higher levels of PSVU have been associated with a range of unfavorable outcomes among adolescents, including poorer psychological well-being, sleep problems, and difficulties in academic and interpersonal adjustment (7–9). These findings suggest that PSVU is associated not only with media-use behavior itself but also with broader aspects of adolescent adjustment. Adolescents with higher PSVU also tend to report heavier academic pressure, weaker academic performance, more exposure to school bullying, poorer parent-child relationships, and less favorable parenting experiences (10). Therefore, identifying psychosocial factors associated with PSVU among adolescents is important for understanding individual differences in problematic short-video use.

Both personal and contextual conditions are associated with PSVU, including psychological characteristics and adverse early experiences. Existing studies report a positive association between emotional maltreatment and adolescent PSVU (11, 12). A global review of meta-analyses reported pooled self-report prevalence estimates of 36.3% for emotional abuse and 18.4% for emotional neglect (13). Among Chinese primary and middle school students, a systematic review and meta-analysis reported pooled prevalence estimates of 30% for emotional abuse and 44% for emotional neglect (14). Emotional maltreatment (EM) refers to non-physical behaviors that undermine an individual’s psychological well-being, personal integrity, or sense of self-worth (15, 16). A systematic review of 68 studies estimated that 19.6% of Chinese children under 18 years of age had experienced emotional abuse (17). Infancy and early adolescence are formative periods for psychological resources; exposure to EM during these stages is associated with poorer mental health (18, 19). More importantly, existing evidence has shown that individuals with childhood EM experiences report higher levels of PSVU than those without such experiences, with a positive association observed between the two variables (20). The compensatory internet use model provides a theoretical basis for understanding this association. According to this model, when emotional support, self-worth, belonging, or other psychological needs are insufficiently met offline, individuals may turn to online environments as an alternative means of psychological compensation (21). EM has also been associated with persistent stress, negative affect, and difficulties in emotion regulation during adolescence (22). In this context, the rapid feedback and attentional distraction provided by short-video platforms may offer adolescents a readily accessible means of temporarily coping with negative experiences. Repeated reliance on this form of online engagement may, in turn, be associated with higher levels of PSVU (23). Taken together, existing studies provide preliminary evidence for a positive association between EM and PSVU. However, previous research has focused primarily on establishing this association, and relatively less is known about the psychological and behavioral factors that may accompany individual differences in PSVU among adolescents with EM experiences. On this basis, the present study first examined the association between EM and adolescent PSVU and proposed that EM would be positively associated with PSVU (H1).

Family emotional experiences constitute an important context for adolescent personality development. As a form of adverse family experience, EM may be particularly relevant to the development of self-related personality characteristics. Previous evidence suggests that childhood maltreatment is associated with narcissistic traits. A three-level meta-analysis showed that child maltreatment was positively associated with both vulnerable and grandiose narcissism, although the overall associations were relatively small (24). Narcissism is commonly conceptualized as encompassing grandiose and vulnerable expressions. Grandiose narcissism is characterized by self-enhancement, dominance, and a need for admiration, whereas vulnerable narcissism is characterized by sensitivity to evaluation, defensiveness, and unstable self-worth (25). Although grandiose and vulnerable narcissism differ in their specific manifestations, both involve heightened self-focus and sensitivity to external recognition. These common characteristics make narcissism relevant to understanding how adverse emotional experiences may be associated with adolescents’ responses to socially evaluative media environments. In the present study, narcissism was examined as a broad trait construct rather than as separate dimension-specific variables. Accordingly, examining narcissism makes it possible to assess whether a self-related personality characteristic statistically accounts for part of the association between EM and PSVU. One possible explanation is that emotionally unavailable, denigrating, or neglectful family interactions may interfere with the development of a stable sense of self-worth. Under such circumstances, adolescents may show greater self-focus, stronger sensitivity to others’ evaluations, or compensatory self-enhancement, all of which are conceptually related to narcissistic traits (26). Consistent with this interpretation, previous studies have also documented associations between parenting experiences and adolescents’ emotional and behavioral development (27). Narcissistic traits may also be relevant to PSVU because the characteristics of short-video platforms correspond closely to individuals’ needs for self-presentation and external recognition. Such platforms provide frequent opportunities for self-presentation, social comparison, and visible interpersonal feedback, which may be particularly salient for adolescents with stronger narcissistic tendencies. Existing empirical evidence provides support for this association. Longitudinal research has shown reciprocal associations between grandiose narcissism and problematic social-networking-site use over an eight-month period (28). In addition, both grandiose and vulnerable narcissism have been associated with problematic TikTok use (29). Taken together, previous studies suggest that EM is associated with narcissistic traits and that narcissism is also associated with problematic digital-media use. However, these relationships have generally been examined separately. Examining EM, narcissism, and PSVU within the same model may therefore provide a more integrated understanding of whether narcissism statistically accounts for part of the association between EM and PSVU. On this basis, the present study hypothesized an indirect statistical association between EM and adolescent PSVU through narcissism (H2).

Physical activity (PA) encompasses bodily movement performed at particular frequencies, intensities, and durations. Extensive theoretical and empirical work associates regular PA with more favorable psychological and physical functioning (30). Self-determination theory identifies autonomy, competence, and relatedness as three basic psychological needs that are important for motivation, development, and well-being (31). Voluntary participation in PA can foster social contact, perceived competence, and achievement. By supporting basic psychological needs and providing opportunities for offline engagement, these experiences may be relevant to lower levels of PSVU. Existing evidence suggests that adolescents exposed to EM or other adverse family circumstances tend to report higher levels of problematic short-video use (32). On this basis, PA may not only be associated with PSVU itself but may also constitute a behavioral condition under which the strength of the EM–PSVU association differs. Previous studies have shown that PA is associated with more favorable psychological functioning and lower levels of problematic internet use (33). More importantly for the present study, PA may provide adolescents with alternative forms of daily engagement outside short-video environments. For adolescents exposed to adverse emotional experiences, greater engagement in PA may provide more opportunities for offline interaction, structured activities, achievement experiences, and social participation. These alternative forms of engagement may reduce reliance on short-video platforms for distraction, feedback, or everyday involvement, providing a possible explanation for why the EM–PSVU association may be weaker at higher levels of PA. Physiological responses accompanying exercise may provide an additional theoretical explanation. Previous research on internet addiction has reported associations of PA with autonomic nervous system function, circulating GDNF (glial cell-derived neurotrophic factor), and glucocorticoid levels (34). These findings provide contextual evidence for understanding the possible relevance of PA, although these physiological processes were not directly assessed in the present study. Among college students, higher levels of PA have been associated with lower levels of short-video addiction (35). More active individuals generally report less susceptibility to addictive behaviors. Experimental findings further suggest that students who regularly engage in aerobic exercise show greater emotional stability and attentional control, alongside lower dependence on short videos (36–38). Previous research has also linked PA with self-control, emotion regulation, and broader psychological functioning. These factors may offer additional theoretical explanations for why the EM–PSVU association differs across levels of PA, although they were not directly measured in the present study. Taken together, existing evidence suggests that PA may be relevant not only to the overall level of PSVU but also to individual differences in the strength of the EM–PSVU association. From this perspective, PA can be considered a behavioral boundary condition of the direct association between EM and PSVU. On this basis, Hypothesis 3 proposes that PA moderates the direct association between EM and adolescent PSVU (H3).

Previous studies have generally examined EM, narcissism, and PA separately in relation to PSVU or related forms of problematic digital-media use. Examining these variables within the same model may therefore provide a more integrated understanding of the different psychological and behavioral factors associated with individual differences in adolescent PSVU. Within the proposed model, EM was treated as the focal exposure associated with PSVU. Narcissism was examined as a personality-related statistical intermediary that may account for part of this association, whereas PA was examined as a behavioral condition under which the remaining direct association between EM and PSVU may vary. Accordingly, the model examines two complementary questions: whether narcissism statistically accounts for part of the EM–PSVU association and whether the remaining direct association differs across levels of PA. Considering these two roles together may provide a more integrated understanding of how personality-related and behavioral factors are associated with variation in PSVU among adolescents reporting EM.

Accordingly, the following hypotheses were proposed:

H1: EM would be positively associated with adolescent PSVU.
H2: An indirect statistical association between EM and adolescent PSVU would be observed through narcissism.
H3: PA would moderate the direct association between EM and adolescent PSVU, such that the association would be weaker at higher levels of PA.

The hypothesized model is presented in **Figure 1**.

**Figure 1:**
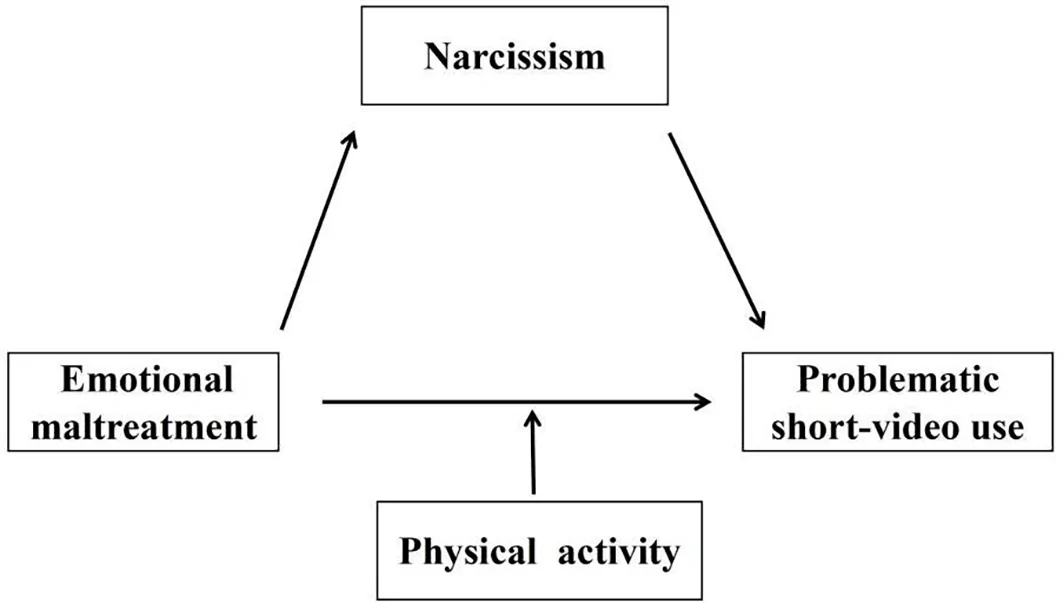
Hypothesized conditional process model.

## 2 Methods

### 2.1 Participants

To evaluate the adequacy of the achieved sample size for the primary interaction analysis, a supplementary power analysis was conducted using G*Power 3.1 within a linear multiple regression framework (fixed model, R² increase). Assuming a small incremental effect size (f² = 0.02), α = 0.05, statistical power of 0.95, one tested predictor (EM × PA), and 20 predictors in the full model, the minimum required sample size was 652. The final analytical sample of 1,622 participants exceeded this requirement. Data collection took place in November 2025 using convenience sampling followed by class-based cluster sampling. Schools that agreed to participate were contacted through convenience sampling, and intact classes were treated as the smallest sampling units. Six schools in Liaoning, Anhui, and Hunan provinces, China, contributed 1,659 recruited students. The study was approved by the Jishou University Biomedical Ethics Committee (JSDX-2025-0107) before data collection. Questionnaires were distributed and retrieved in classrooms. Before participation, students were informed of the study purpose and procedures, the voluntary nature of participation, protections for anonymity and confidentiality, and the intended use of their data. For participants younger than 18 years, written informed consent was obtained from their parents or legal guardians, and assent was obtained from the participants themselves before participation. Participants aged 18–19 years provided written informed consent directly. Participants were informed that they could decline to answer any question or discontinue participation at any time without penalty. Because the questionnaire included potentially sensitive items concerning emotional maltreatment, privacy and confidentiality were emphasized during classroom administration, and participants were instructed to complete the questionnaire independently without discussing their responses with classmates. Participants who experienced discomfort were permitted to discontinue the questionnaire. If a participant directly disclosed information suggesting possible ongoing maltreatment or requested assistance, the situation was handled in accordance with the safeguarding procedures approved for the study, including referral to appropriate school-based or professional support when necessary. The study was conducted in accordance with the local legislation and institutional requirements. Before analysis, all questionnaires were screened according to predefined data-quality criteria. Questionnaires were excluded if they contained any unanswered item, if completion time deviated by more than three standard deviations from the sample mean, or if they showed clear straightlining, characterized by repeated selection of the same response option across multiple consecutive items or substantively distinct scales. Of the 1,659 questionnaires collected, 37 were excluded during screening, including 12 because of unanswered items, 15 because of abnormal completion times, and 10 because of clear straightlining. The final analytical sample therefore comprised 1,622 participants. Because questionnaires containing unanswered items were excluded at the screening stage, the final analytical dataset contained no item-level missing data. The analytical sample comprised 1,622 adolescents, including 772 males and 850 females, aged 10–19 years (*M* = 14.63, *SD* = 1.66). By educational stage, the mean age was 11.85 years (*SD* = 1.15) for primary school students, 14.53 years (*SD* = 0.93) for junior high school students, and 16.26 years (*SD* = 0.92) for senior high school students. Detailed demographic characteristics of the participants are presented in **Table 1**.

**Table 1:** Participant characteristics (N = 1,622).

Variable	Category	n	%
Gender	Male	772	47.6
	Female	850	52.4
Age (years)	10–19	1622	100
Grade	Primary school	229	14.1
	Junior high school	939	57.9
	Senior high school	454	28.0
Only child	Yes	377	23.2
	No	1245	76.8
Residence	Rural	135	8.3
	Urban	1487	91.7
Mother’s education	≤Primary school	304	18.7
	Secondary school	747	46.1
	University	226	13.9
	≥Graduate	30	1.8
	Unknown	315	19.4
Father’s education	≤Primary school	242	14.9
	Secondary school	783	48.3
	University	260	16.0
	≥Graduate	35	2.2
	Unknown	302	18.6
Perceived economic status	Low (1–3)	179	11.0
	Medium (4–7)	1290	79.5
	High (8–10)	153	9.4
Children left behind	Yes	487	30.0
	No	1135	70.0

### 2.2 Measures

#### 2.2.1 Emotional maltreatment

The full Childhood Trauma Questionnaire-Short Form (CTQ-SF), developed and validated by Bernstein et al. (39) and subsequently adapted and evaluated in Chinese populations by Zhao et al. (40), served as the parent instrument from which the abbreviated four-item composite used in the present study was derived. Previous psychometric studies have supported the reliability and validity of the Chinese CTQ-SF in adolescent populations, including a large nationwide sample of Chinese adolescents aged 12–18 years (41, 42). The CTQ-SF has also been applied in recent research involving a large sample of Chinese adolescents aged 10–19 years (43). The original CTQ-SF asks respondents to report experiences occurring “when I was growing up.” The present study used an abbreviated four-item measure of EM derived from the CTQ-SF, comprising two items from the emotional abuse subscale and two positively worded items from the emotional neglect subscale. Specifically, the four items assessed whether, during childhood (1), family members had said insulting or hurtful things to the participant (2); the participant felt valued, loved, and supported by family members; (3) the participant felt that someone in the family hated them and caused emotional suffering or psychological harm; and (4) family members cared for one another and maintained close relationships. The second and fourth items were reverse scored before calculation of the composite score so that higher scores consistently represented greater emotional maltreatment. Given that the age range of the present sample extended beyond that of the available Chinese psychometric validation samples and that an abbreviated four-item composite was used, additional psychometric analyses were conducted in the present sample to examine its structural coherence and internal consistency. The four-item assessment was specified before the present analyses and was not derived through data-driven item selection in the current sample. Its use was informed by previous research employing abbreviated CTQ-SF-derived measures comprising two emotional abuse items and two emotional neglect items. For example, Yan et al. (44) used a comparable four-item assessment strategy to operationalize childhood emotional maltreatment. Accordingly, the present four-item composite was used as a brief operational measure of EM rather than as a substitute for the complete emotional abuse and emotional neglect subscales of the CTQ-SF. In the present study, the resulting composite score was operationally defined as EM, with higher scores indicating higher levels of EM-related adverse emotional experiences. Responses were rated on a five-point Likert scale from 1 (never) to 5 (always), yielding total scores from 4 to 20.

To examine the structural coherence of the four-item measure in the present sample, the full sample was randomly divided into two non-overlapping subsamples of equal size (n = 811 each). Exploratory factor analysis was conducted in the first subsample. The Kaiser–Meyer–Olkin value was 0.752, and Bartlett’s test of sphericity was statistically significant, χ² (6) = 908.38, p < 0.001. A single factor with an eigenvalue greater than 1 (2.416) was identified, accounting for 60.41% of the total variance. Factor loadings ranged from 0.686 to 0.828. Confirmatory factor analysis was subsequently conducted in the second subsample using the weighted least squares mean- and variance-adjusted estimator, with the four items treated as ordered categorical indicators. A residual covariance between the two emotional neglect indicators was prespecified before model estimation on theoretical and measurement grounds. Both indicators originated from the emotional neglect domain, were positively worded and subsequently reverse scored, and shared closely related content concerning family emotional care and support. The one-factor model showed high CFI and TLI values and a low SRMR, whereas the RMSEA was elevated, χ²(1) = 9.135, p = 0.003, CFI = 0.997, TLI = 0.984, RMSEA = 0.100, 90% CI [0.048, 0.164], and SRMR = 0.008. Given the very low model degrees of freedom (df = 1), the RMSEA was interpreted cautiously. Standardized factor loadings ranged from 0.671 to 0.895. After reverse scoring the two emotional neglect items, Cronbach’s α for the four-item measure was 0.797. Measurement invariance across educational stages was further examined using multigroup CFA with the WLSMV estimator. The configural model showed high CFI and TLI values and a low SRMR (CFI = 0.997, TLI = 0.981, SRMR = 0.009), although the RMSEA was elevated (RMSEA = 0.104) and was interpreted cautiously given the very low model degrees of freedom (df = 3). A more constrained model imposing equality constraints on factor loadings and thresholds showed good fit, χ²(31) = 46.394, CFI = 0.997, TLI = 0.998, RMSEA = 0.030, and SRMR = 0.017. The WLSMV DIFFTEST indicated that the equality constraints did not significantly worsen model fit, Δχ²(28) = 35.279, p = 0.162, supporting measurement invariance of the abbreviated four-item EM measure across primary-, junior-high-, and senior-high-school students.

#### 2.2.2 Narcissism

Narcissistic traits were assessed using the Chinese version of the 15-item Five-Factor Narcissism Inventory–Super-Short Form (FFNI-SSF). The original FFNI-SSF was developed by Packer West et al. (45), and the Chinese version used in the present study was translated and psychometrically evaluated by Chen et al. (46). The scale includes characteristics relevant to both grandiose and vulnerable expressions of narcissism. Each item was rated on a five-point Likert scale ranging from 1 (strongly disagree) to 5 (strongly agree). In the present study, the research hypothesis concerned narcissism as a broad self-related personality characteristic rather than the differential roles of grandiose and vulnerable narcissism. Accordingly, item scores were summed to obtain an overall narcissistic-trait composite, with higher scores indicating higher overall levels of narcissistic traits. This use of the total score does not imply that grandiose and vulnerable narcissism are conceptually equivalent; rather, the composite was used to represent broad narcissistic tendencies within the scope of the present model. Cronbach’s α for the overall score in the present adolescent sample was 0.747.

#### 2.2.3 Physical activity

PA was measured with the Physical Activity Rating Scale revised by Liang et al. (1994). The scale includes three items assessing exercise intensity, duration, and frequency on five-point Likert response options (47). In the present study, physical activity was operationalized as exercise participation quantified by exercise intensity, duration, and frequency using this scale. The composite score was computed as PA score = intensity × (duration − 1) × frequency. Possible scores ranged from 0 to 100, with higher values representing higher PA.

#### 2.2.4 Problematic short-video use

Problematic short-video use was assessed using the Problematic Short Video Media Use Scale developed by Mao et al. (48). The scale has been used in Chinese adolescent populations, including secondary-school students (49) and adolescents aged 10–19 years across primary-, junior-high-, and senior-high-school stages (50). Psychometric research has further supported measurement equivalence of the scale across high-school and university-student samples (51). In the present study, the scale was used to assess the severity of problematic short-video use rather than to establish a clinical diagnosis of addiction. The scale consists of 13 items across three dimensions. Participants responded on a five-point Likert scale ranging from 1 (completely disagree) to 5 (completely agree), with higher summed scores indicating higher levels of problematic short-video use. Cronbach’s α was 0.915 in the present sample.

#### 2.2.5 Sociodemographic variables

Sociodemographic information included gender, age, educational stage, only-child status, residence, parental education, perceived family economic status, and left-behind status. Gender was coded as 1 = male and 2 = female; only-child status as 1 = yes and 2 = no; residence as 1 = rural and 2 = urban; and left-behind status as 1 = yes and 2 = no. Age was entered as a continuous variable. For data entry and descriptive reporting, educational stage was coded as 1 = primary school, 2 = junior high school, and 3 = senior high school. In the regression analyses, educational stage was treated as a categorical covariate using indicator coding, as described in the Data Processing and Analysis section. For data entry and descriptive reporting, maternal and paternal educational attainment was classified as 1 = primary school or below, 2 = secondary school, 3 = university, 4 = graduate level or above, and 5 = unknown. The “unknown” category denoted cases in which adolescents were unable to report the corresponding parent’s highest level of educational attainment. Perceived family economic status was rated on a 10-point scale, with higher scores indicating better perceived family economic status. For descriptive presentation, these scores were categorized as low (1–3), medium (4–7), or high (8–10), whereas the original 1–10 score was retained as a continuous covariate in the regression analyses.

### 2.3 Data processing and analysis

SPSS 27.0 together with PROCESS macro version 4.1 was used for data processing, descriptive statistics, correlation analyses, exploratory factor analysis, and conditional process analyses, whereas Mplus 8.3 was used for confirmatory factor analysis and measurement-invariance analyses. Confirmatory factor analysis of the abbreviated four-item EM measure was conducted in an independent subsample of 811 participants. The four items were specified as ordered categorical indicators and estimated using the weighted least squares mean- and variance-adjusted (WLSMV) estimator. A residual covariance between the two emotional neglect indicators was prespecified before model estimation on theoretical and measurement grounds. Model fit was evaluated using the comparative fit index (CFI), Tucker–Lewis index (TLI), root mean square error of approximation (RMSEA), and standardized root mean square residual (SRMR). Measurement invariance across educational stages was examined using multigroup CFA with the WLSMV estimator. A configural model was compared with a more constrained model in which factor loadings and thresholds were held equal across primary-, junior-high-, and senior-high-school students, and nested models were compared using the DIFFTEST procedure. Internal consistency was assessed using Cronbach’s α after reverse scoring the two emotional neglect items. Common method bias was examined first; the prespecified criterion required the first factor to account for less than 40% of total variance (52). Descriptive statistics summarized participant characteristics and the main study variables. Distributional characteristics of the continuous variables were additionally examined using the observed range, median, interquartile range, skewness, and kurtosis. Correlation analysis was used to quantify bivariate associations. All continuous variables entered into the PROCESS models were standardized as z scores before analysis. Specifically, EM and PA were standardized before construction of the EM × PA interaction term. The analyses were conducted in two steps. First, PROCESS Model 4 was used to quantify the total, direct, and indirect statistical associations between EM and PSVU through narcissism. Second, PROCESS Model 5 was used to examine whether PA moderated the remaining direct association between EM and PSVU while retaining narcissism as the intermediate variable. The indirect statistical association was evaluated using 5,000 bootstrap samples with 95% bootstrap confidence intervals. Conditional direct associations were subsequently probed within PROCESS Model 5 and reported with 95% confidence intervals. Because *M − 1 SD* for PA fell below its observed range, probing the interaction at that value would have required extrapolation beyond the empirical range of the data. Therefore, relatively low and high levels of PA were represented by the 16th and 84th percentiles of the observed PA distribution, respectively (53). For ease of interpretation, the PA values used to probe and plot the interaction are reported on the original scale; P16 = 0, *M* = 20.73, and P84 = 40 corresponded to standardized values of −0.857, 0, and 0.797, respectively. PA was retained as a continuous variable throughout the moderation analysis. Sensitivity analyses were additionally conducted by educational stage to examine whether the moderation pattern was consistent across primary, junior high, and senior high school students. Gender, age, educational stage, only-child status, residence, maternal education, paternal education, perceived family economic status, and left-behind status were included as sociodemographic covariates in the primary analyses. Age and the original 1–10 perceived family economic status score were included as continuous covariates. Gender, only-child status, residence, and left-behind status were entered using their binary coding. Educational stage was modeled as a categorical covariate using indicator coding, with primary school as the reference category and separate indicators representing junior high school and senior high school. Maternal and paternal educational attainment was modeled as categorical covariates using indicator coding. For each parent, primary school or below served as the reference category, with separate indicators representing secondary school, university, graduate level or above, and unknown. Statistical significance was defined as α = 0.05. To facilitate reproducibility, the complete SPSS and PROCESS syntax corresponding to the reported primary analyses is provided in **Supplementary Material S1**.

## 3 Results

### 3.1 Common method bias test

Six factors with eigenvalues greater than 1 emerged from the common method bias analysis. The first factor explained 17.62% of the total variance, below the 40% threshold. The analysis therefore provided no indication of serious common method bias.

### 3.2 Descriptive analysis

The analytical sample included 1,622 adolescents: 772 males (47.6%) and 850 females (52.4%), aged 10 to 19 years. Junior high school students accounted for the largest proportion (57.9%), followed by senior high school students (28.0%) and primary school students (14.1%). Only children numbered 377 (23.2%), whereas 1,245 participants (76.8%) had siblings. Most respondents resided in urban areas (91.7%). Secondary school was the most commonly reported education level for both mothers and fathers. Most participants rated family economic status as medium (79.5%), with smaller groups in the low- and high-income categories. The sample included 30.0% left-behind children and 70.0% non-left-behind children. The PA composite showed a positively skewed distribution, with scores ranging from 0 to 100, a median of 12.00 (IQR = 4.00–30.00), skewness of 1.679, and kurtosis of 2.467.

### 3.3 Correlation analysis

**Table 2** presents the descriptive statistics and bivariate correlations among the main study variables. EM was positively correlated with PSVU (*r* = 0.292, *p* < 0.001) and narcissism (*r* = 0.187, *p* < 0.001). Narcissism was also positively correlated with PSVU (*r* = 0.249, *p* < 0.001), whereas PA was negatively correlated with PSVU (*r* = −0.163, *p* < 0.001). **Figure 2** provides a visual overview of the direction and relative magnitude of these correlations.

**Table 2:** Descriptive statistics and correlations among the main study variables.

Variables	1	2	3	4
1 EM	-			
2 Narcissism	0.187***	-		
3 PSVU	0.292***	0.249***	-	
4 PA	-0.132***	-0.003	-0.163***	-
*M*	9.47	41.09	20.71	20.73
*SD*	3.63	9.07	8.04	24.18

*** *p* < 0.001.

**Figure 2:**
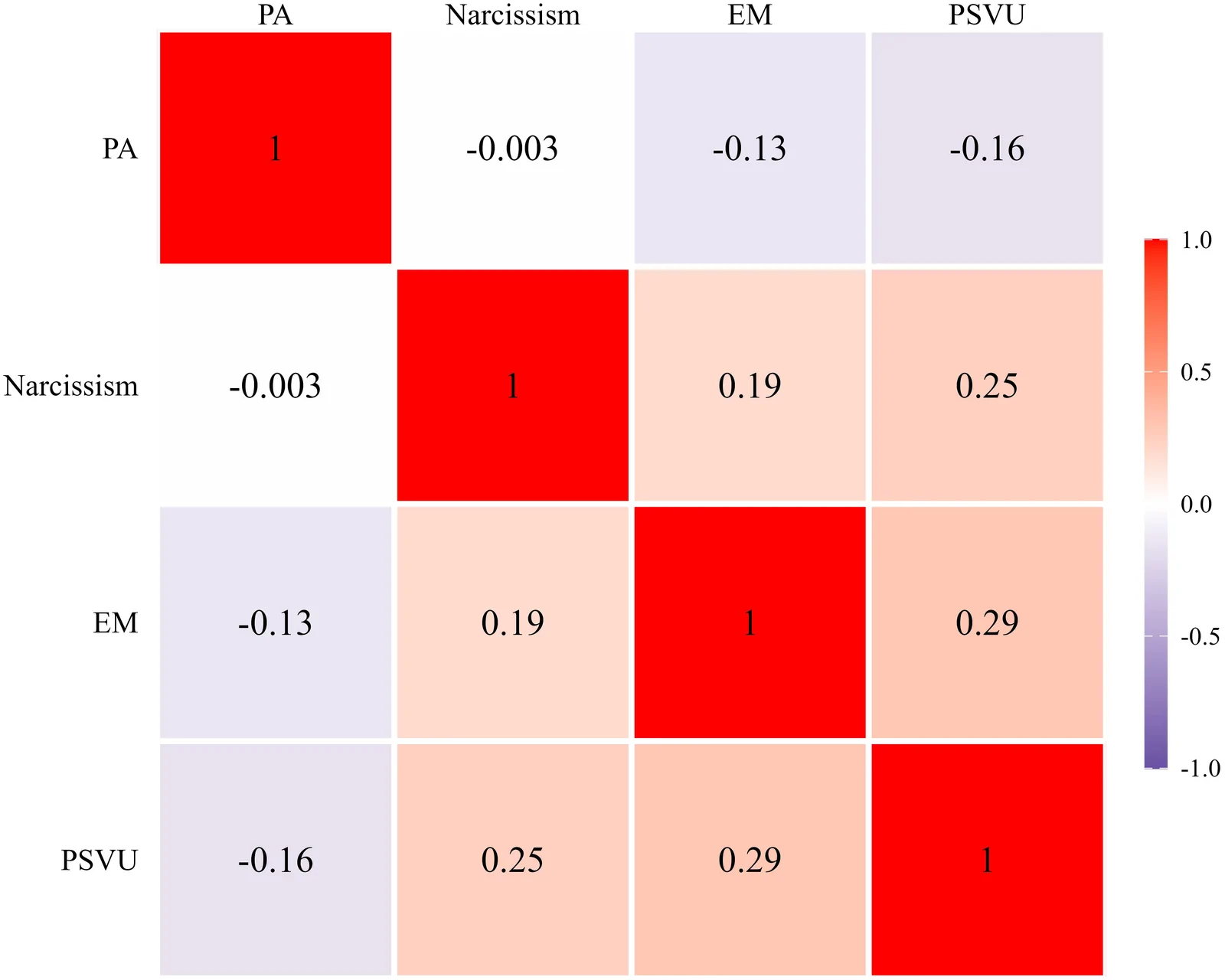
Correlation heatmap of the main study variables.

### 3.4 Indirect statistical association between EM and PSVU through narcissism

The analysis of the indirect statistical association showed that, after controlling for the sociodemographic covariates, EM was positively associated with narcissism and PSVU, and narcissism was positively associated with PSVU after controlling for EM. Bootstrap analysis indicated that the indirect statistical association between EM and PSVU through narcissism was statistically significant, as the 95% confidence interval did not include zero. The direct association between EM and PSVU remained statistically significant after narcissism was included in the model, indicating that narcissism statistically accounted for part of the association between EM and PSVU. Thus, H2 was supported. Detailed regression results are presented in **Table 3**. The total, direct, and indirect statistical associations are presented in **Table 4**. The corresponding model is illustrated in **Figure 3**.

**Table 3:** Regression models for the indirect statistical association through narcissism.

Outcome variables	Explanatory variables	*β*	*SE*	*t*	*R²*	*F(df1, df2)*
PSVU	EM	0.268	0.023	11.576***	0.185	F(17, 1604) = 21.422***
Narcissism	EM	0.192	0.025	7.718***	0.063	F(17, 1604) = 6.291***
PSVU	EM	0.236	0.023	10.166***	0.211	F(18, 1603) = 23.869***
	Narcissism	0.168	0.023	7.317***		

All models were adjusted for the sociodemographic covariates specified in the Methods section. *** *p* < 0.001.

**Table 4:** Total, direct, and indirect statistical associations between EM and PSVU.

Association type	Estimate	*SE*	95% CI	Proportion of total association
Total association	0.268	0.023	[0.223, 0.313]	
Direct association	0.236	0.023	[0.190, 0.281]	88.01%
Indirect statistical association	0.032	0.007	[0.019, 0.048]	11.99%

The indirect statistical association was evaluated using 5,000 bootstrap samples and a 95% bootstrap confidence interval.

**Figure 3:**
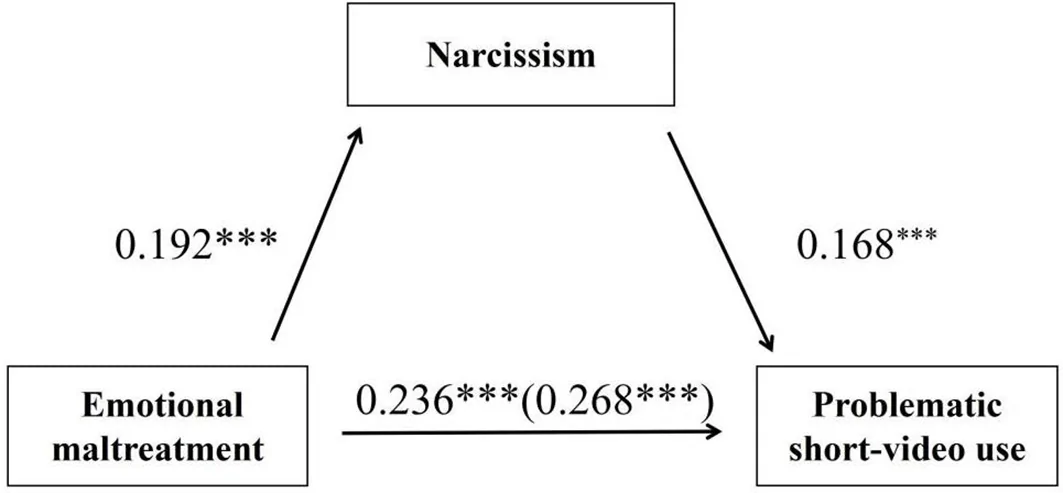
Indirect statistical association through narcissism. *** p < 0.001.

Values are standardized regression coefficients adjusted for sociodemographic covariates. For the EM–PSVU path, the value outside parentheses is the direct association, and the value in parentheses is the total association. *** *p* < 0.001.

### 3.5 Moderation of the direct association between EM and PSVU

After PA and the EM × PA interaction were entered into the model, EM remained positively associated with PSVU, whereas PA was negatively associated with PSVU. The EM × PA interaction was statistically significant, indicating that PA moderated the direct association between EM and PSVU. Conditional effect analyses showed that the positive association between EM and PSVU remained statistically significant at low (P16 = 0), mean (M = 20.73), and high (P84 = 40) levels of PA, while the magnitude of the association progressively decreased as PA increased (**Table 5**; **Figure 4**). For graphical presentation, low and high EM were represented by *M − 1 SD* and *M + 1 SD*, respectively. Because *M − 1 SD* for PA fell below its observed range, low and high PA were represented by the 16th and 84th percentiles of the observed PA distribution, respectively (corresponding to raw PA scores of 0 and 40). This percentile-based approach ensured that the plotted PA values remained within the observed range. EM and PA were retained as continuous variables in all statistical analyses. Complete regression estimates are provided in **Table 6**, while **Figure 5** visually summarizes the conditional process model. Sensitivity analyses stratified by educational stage yielded negative EM × PA interaction estimates among primary-school students (β = −0.089, SE = 0.049, 95% CI [−0.186, 0.008], *p* = 0.071), junior-high-school students (β = −0.074, *SE* = 0.031, 95% CI [−0.135, −0.013], *p* = 0.018), and senior-high-school students (β = −0.078, *SE* = 0.045, 95% CI [−0.166, 0.011], *p* = 0.086). Although the interaction estimates were negative and similar in magnitude across all three educational stages, the confidence intervals included zero for the primary-school and senior-high-school subgroups, whereas the interaction was statistically significant in the junior-high-school subgroup.

**Table 5:** Conditional direct associations between EM and PSVU at different levels of PA.

PA Levels	Estimate	*SE*	*t*	Lower 95% CI	Upper 95% CI
Low (P16 = 0)	0.286	0.030	9.454***	0.227	0.345
Mean (M = 20.73)	0.230	0.023	9.921***	0.184	0.275
High (P84 = 40)	0.178	0.029	6.118***	0.121	0.235

Low and high PA correspond to the 16th and 84th percentiles of the observed PA distribution, respectively. *** *p* < 0.001.

**Figure 4:**
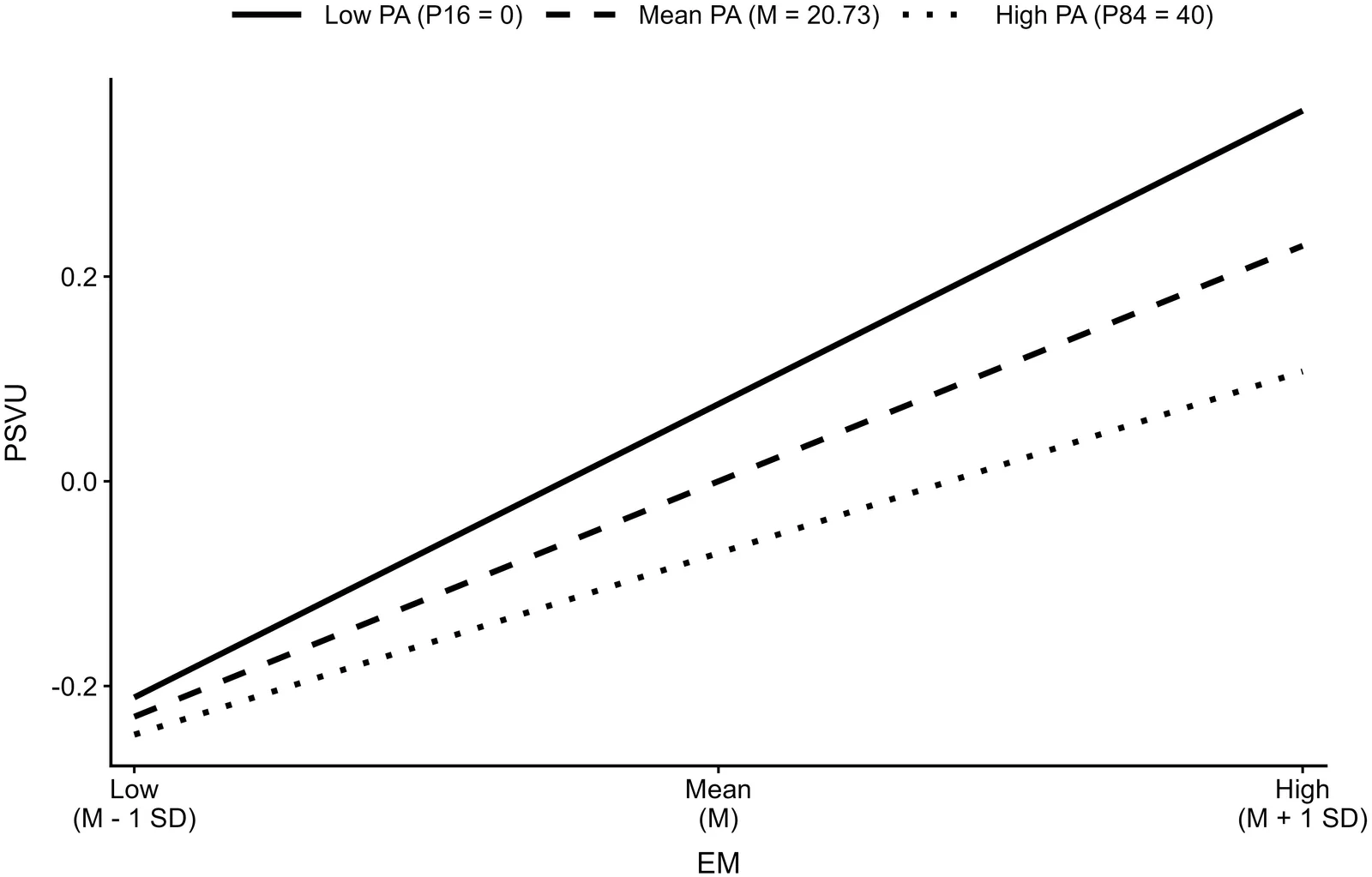
Moderating role of PA in the association between EM and PSVU.

**Table 6:** Regression results for the conditional process model.

Outcome variable	Explanatory variable	*β*	*SE*	*t*	*R²*	*F(df1, df2)*
Narcissism	EM	0.192	0.025	7.718***	0.063	F(17, 1604) = 6.291***
PSVU	EM	0.230	0.023	9.921***	0.221	F(20, 1601) = 22.649***
	Narcissism	0.171	0.023	7.479***		
	PA	-0.088	0.024	-3.657***		
	EM × PA	-0.066	0.022	-2.934**		

** *p* < 0.01; *** *p* < 0.001.

**Figure 5:**
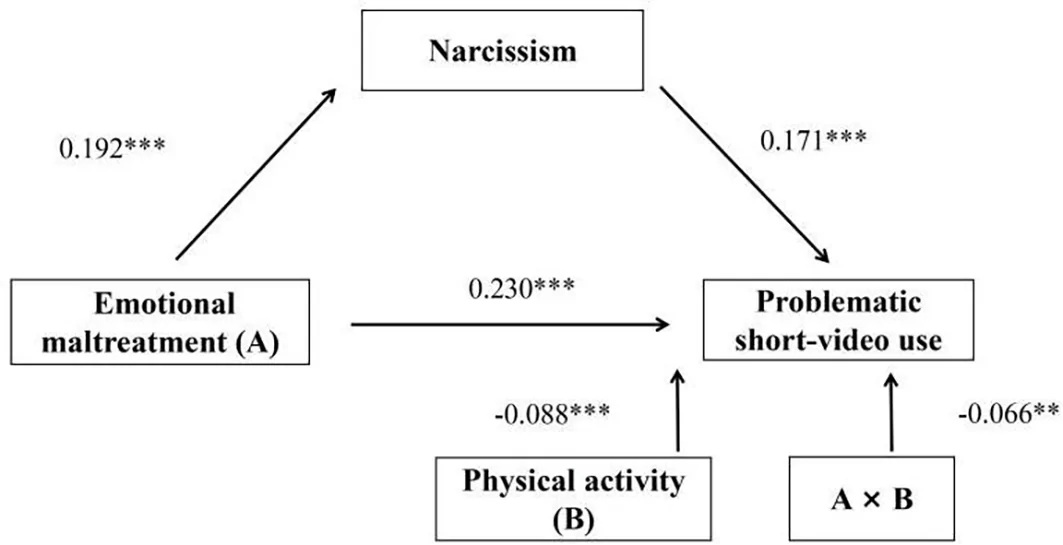
Conditional process model of the associations among emotional maltreatment, narcissism, physical activity, and problematic short-video use. ** p < 0.01; *** p < 0.001.

Values are standardized regression coefficients adjusted for the sociodemographic covariates specified in the Methods section. ** *p* < 0.01, *** *p* < 0.001.

Low and high EM represent *M − 1 SD* and *M + 1 SD*, respectively. Low, mean, and high PA correspond to P16 = 0, M = 20.73, and P84 = 40, respectively. The y-axis represents model-predicted standardized PSVU scores.

## 4 Discussion

The present study yielded three main findings. First, EM was positively associated with adolescent PSVU. Second, an indirect statistical association between EM and PSVU was observed through narcissism, although this indirect component accounted for only a modest proportion of the total association. Third, PA moderated the remaining direct association between EM and PSVU, such that the positive association was weaker at higher levels of PA. Taken together, these findings suggest that the association between adverse emotional experiences and adolescent PSVU may be understood in relation to both self-related personality characteristics and physical activity engagement. Given the cross-sectional design, however, these patterns should be interpreted as statistical associations rather than as evidence of temporal or causal processes.

The first main finding was that EM was positively associated with adolescent PSVU, supporting Hypothesis 1 and broadly aligning with previous empirical evidence (54, 55). Several theoretically plausible explanations may help to understand this association. Emotion-regulation theory suggests that maltreated children may receive fewer opportunities to acquire adaptive regulation skills and less parental support during distress (56, 57). Their family environments may consequently offer limited examples of constructive emotional expression, reassurance, and problem solving. Adolescents in such circumstances may suppress distress or turn to readily accessible mood-regulation activities, including excessive short-video use (58, 59). The novelty, immediate feedback, and brief attentional escape offered by short videos may be particularly appealing when offline emotional regulation is difficult. Individuals exposed to EM often report lower self-worth and poorer social functioning than peers without maltreatment histories (60). EM is also negatively associated with self-esteem and positively associated with emotional and behavioral difficulties (61). Difficulties in emotion regulation may also be relevant to problematic short-video engagement, as maladaptive cognitive emotion-regulation strategies have been associated with higher levels of short-video addiction among children and adolescents (62). Further evidence has linked childhood emotional maltreatment with problematic social media use among adolescents (18, 63). The current finding is therefore consistent with existing research and positions EM as a meaningful psychosocial correlate of PSVU. It should also be noted that the observed association may partly reflect other family, emotional, or interpersonal conditions that were not measured in the present study. Moreover, the cross-sectional design does not permit conclusions regarding whether EM temporally preceded or causally contributed to PSVU.

An indirect statistical association between EM and PSVU through narcissism was observed, supporting Hypothesis 2. The indirect statistical association was modest in magnitude (0.032), accounting for approximately 11.99% of the total association. This finding suggests that narcissism may represent one self-related psychological correlate linking EM with PSVU, although its explanatory contribution appears limited. A possible interpretation is that adverse emotional experiences within the family may be relevant to narcissistic traits. Consistent with this interpretation, meta-analytic evidence has shown positive associations between child maltreatment and both vulnerable and grandiose narcissism (24). Short-video platforms, in turn, provide frequent opportunities for self-presentation, social comparison, and visible interpersonal feedback, which may be particularly salient for adolescents with stronger narcissistic tendencies. Consistent with this interpretation, previous research has documented a positive association between narcissism and PSVU (29). Nevertheless, the relatively small indirect association indicates that narcissism accounts for only a limited portion of the EM–PSVU association, suggesting that other psychological, interpersonal, and contextual factors may also be involved. In addition, because the present study used the overall FFNI-SSF score, the observed association cannot be attributed specifically to grandiose or vulnerable narcissism. Given the cross-sectional design, the indirect statistical association should be interpreted as a statistical pattern rather than evidence of temporal ordering or a causal mediating process.

The third main finding was that PA moderated the direct association between EM and PSVU in the overall sample, with the positive association weakening as PA increased, providing support for H3 at the overall-sample level. However, although the EM × PA interaction was negative across all three educational stages, it reached statistical significance only among junior high school students. Although statistical significance was reached only in the junior-high-school subgroup, the interaction estimates were similar in direction and magnitude across the three educational stages. Therefore, these subgroup findings should not be interpreted as evidence of statistically significant differences in the moderating effect across educational stages. The interaction estimate was modest in magnitude (*β* = −0.066), reflecting limited variation in the strength of the EM–PSVU association across levels of PA. Given that within-class and within-school dependence could not be modeled and that the overall EM × PA interaction was modest in magnitude, the statistical significance of this moderation finding should be interpreted cautiously and requires replication in studies that retain cluster identifiers and appropriately account for clustered observations. This finding is broadly consistent with previous evidence showing a negative association between PA and adolescent PSVU (64). One possible interpretation is that greater engagement in PA may provide adolescents with more opportunities for structured offline activity, sustained behavioral engagement, and self-regulatory practice. At the same time, time devoted to PA may reduce opportunities for prolonged short-video use and provide alternative sources of social interaction and achievement outside digital environments. From this perspective, PA may be better understood as a behavioral condition associated with variation in the EM–PSVU relationship rather than as a factor that substantially offsets the adverse consequences associated with EM. This interpretation should nevertheless remain cautious because the present study did not directly assess the psychological processes, motivation, quality, or social context accompanying PA. Accordingly, the observed interaction does not demonstrate a specific protective mechanism of PA. Moreover, the cross-sectional design does not establish whether greater PA preceded a weaker EM–PSVU association or whether adolescents with lower PSVU were more likely to engage in PA.

Using a Chinese adolescent sample, this study examined EM, narcissism, PSVU, and PA within a single analytical framework that integrated adverse developmental experience, personality, and problematic media use. Narcissism statistically accounted for part of the association between EM and PSVU, and the strength of that association differed by PA. These findings may inform family- and school-based efforts addressing adolescents’ emotional experiences, self-related adjustment, media-use habits, and physical activity engagement, while remaining consistent with the observational nature of the present study. Several limitations require attention. First, participants were recruited from selected Chinese regions through convenience sampling followed by cluster sampling, and students were nested within classes and schools. School and class identifiers were not retained in the analytical dataset, so within-cluster dependence could not be modeled. Standard errors may therefore have been underestimated, potentially increasing the statistical significance of some associations. Future research should preserve cluster identifiers and use cluster-robust standard errors or multilevel models. Second, every variable was assessed at one time point. Significant direct, indirect, and conditional associations do not reveal temporal precedence, reciprocal relations, or directionality. Longitudinal and prospective studies are needed to determine how the variables change together and how their ordering unfolds over time. Alternative models may also clarify whether narcissism operates as an intermediary, a correlate, or part of a reciprocal relation. Third, retrospective self-report measures are vulnerable to recall error, shared response tendencies, and socially desirable responding, which may reduce objectivity and precision. Reports of childhood EM may be particularly influenced by memory and current emotional state. Future studies could combine self-report measures with behavioral indicators, parent, teacher, or peer reports, and physiological measures. Fourth, EM was assessed using an abbreviated four-item measure derived from the CTQ-SF, including two emotional abuse items and two reverse-scored emotional neglect items. Although supplementary psychometric analyses supported a coherent one-factor structure, acceptable internal consistency, and measurement invariance across educational stages, the abbreviated four-item measure does not fully capture the broader construct domain of emotional maltreatment represented by the complete emotional abuse and emotional neglect subscales of the CTQ-SF. Therefore, findings involving EM should be interpreted with appropriate caution. Future studies should employ more comprehensive and fully validated measures of EM to capture the broader construct domain more adequately. Fifth, the present analysis used the overall FFNI-SSF score as a broad composite of narcissistic traits and did not separately estimate grandiose and vulnerable narcissism. Accordingly, the observed indirect statistical association cannot be attributed to either specific dimension. In addition, the PROCESS analyses were based on observed composite scores rather than latent variables, and measurement error was therefore not explicitly modeled. This may have affected the magnitude and precision of the estimated direct, indirect, and interaction associations. Future studies may employ dimension-specific measures and latent-variable approaches to examine the potentially distinct roles of grandiose and vulnerable narcissism while explicitly accounting for measurement error.

## 5 Conclusion

This cross-sectional study found a positive association between adolescent EM and PSVU. An indirect statistical association was observed through narcissism, while PA moderated the direct association between EM and PSVU. These findings may inform family- and school-based efforts concerning adolescents’ emotional needs, self-related adjustment, healthy media-use habits, and regular physical activity.

## Data Availability

The raw data supporting the conclusions of this article will be made available by the authors, without undue reservation.
