# Supplementary material for: Emotional maltreatment and problematic short-video use among adolescents: an indirect association through narcissism and moderation of the direct association by physical activity
Source: Front Psychiatry. 2026 Sep 25;17:1958164. doi: 10.3389/fpsyt.2026.1958164 (PMC13650740; doi:10.3389/fpsyt.2026.1958164)
Supplement: Supplementary file 1 [file SupplementaryFile1.doc]

Supplementary Material S1

Full SPSS and PROCESS Syntax for the Primary Analyses

*=============================================================.

* 1. Indicator coding of categorical covariates.

* Reference category for educational stage:

* primary school (grade = 1).

*=============================================================.

COMPUTE grade_junior = (grade = 2).

COMPUTE grade_senior = (grade = 3).

* Maternal education.

* Reference category: primary school or below (mother = 1).

COMPUTE mother_secondary = (mother = 2).

COMPUTE mother_university = (mother = 3).

COMPUTE mother_graduate = (mother = 4).

COMPUTE mother_unknown = (mother = 5).

* Paternal education.

* Reference category: primary school or below (father = 1).

COMPUTE father_secondary = (father = 2).

COMPUTE father_university = (father = 3).

COMPUTE father_graduate = (father = 4).

COMPUTE father_unknown = (father = 5).

EXECUTE.

*=============================================================.

* 2. Standardization of continuous variables.

*=============================================================.

DESCRIPTIVES VARIABLES = catot narc dsp pa age econ

/SAVE.

*=============================================================.

* 3. PROCESS Model 4.

* X = Emotional maltreatment.

* M = Narcissism.

* Y = Problematic short-video use.

*=============================================================.

PROCESS vars =

Zcatot Znarc Zdsp

gender Zage grade_junior grade_senior

only resid

mother_secondary mother_university mother_graduate mother_unknown

father_secondary father_university father_graduate father_unknown

Zecon left

/y = Zdsp

/x = Zcatot

/m = Znarc

/model = 4

/cov =

gender Zage grade_junior grade_senior

only resid

mother_secondary mother_university mother_graduate mother_unknown

father_secondary father_university father_graduate father_unknown

Zecon left

/boot = 5000

/total = 1.

*=============================================================.

* 4. PROCESS Model 5.

* Narcissism is the mediator.

* PA moderates the direct EM -> PSVU association.

*=============================================================.

PROCESS vars =

Zcatot Znarc Zdsp Zpa

gender Zage grade_junior grade_senior

only resid

mother_secondary mother_university mother_graduate mother_unknown

father_secondary father_university father_graduate father_unknown

Zecon left

/y = Zdsp

/x = Zcatot

/m = Znarc

/w = Zpa

/model = 5

/cov =

gender Zage grade_junior grade_senior

only resid

mother_secondary mother_university mother_graduate mother_unknown

father_secondary father_university father_graduate father_unknown

Zecon left

/boot = 5000

/wmodval = -0.857521, 0, 0.797031.
